# Strengths and Limits of Psychodynamic Intervention Rating Scale (PIRS) in Capturing Psychotherapeutic Micro‐Process: A Systematic Review

**DOI:** 10.1002/cpp.70073

**Published:** 2025-05-01

**Authors:** Arianna Palmieri, Valentina Cimmino Picone, Alessandro Gennaro, Davide Ruffin, Valeria Capuani, Johann Roland Kleinbub

**Affiliations:** ^1^ Department of Philosophy, Sociology, Education and Applied Psychology (FISSPA) University of Padua Padua Italy; ^2^ Department of Psychology and Health Sciences Pegaso Telematic University Naples Italy

**Keywords:** expressive–supportive continuum, PIRS, Psychodynamic Intervention Rating Scale, psychological interventions, verbal intervention

## Abstract

**Background:**

The Psychodynamic Intervention Rating Scale (PIRS) stands out as one of the most widely utilised coding systems aimed at categorising micro‐process from psychotherapeutic dialogue due to its feasibility and adherence to the expressive–supportive continuum. Despite this, no comprehensive analysis of its application in the literature has been published. This systematic review aims to examine the state of the art, strengths, limitations and future perspectives of such a coding tool for verbatim transcripts.

**Methods:**

Following Preferred Reporting Items for Systematic Reviews and Meta‐Analyses (PRISMA) guidelines, a systematic search in databases (PsycINFO, Scopus, PubMed and Web of Science) covering publications from the PIRS's first occurrence in the scientific literature in 1992 to 2024 yielded 22 publications eligible for review. Three independent reviewers screened the studies and extracted data regarding PIRS applications and key findings.

**Results:**

The studies examined revealed that PIRS can reliably classify interventions across various therapeutic approaches, surpassing its psychodynamic origins. It demonstrated peculiar efficacy in understanding therapeutic alliance mechanisms and patients' responses according to their typical defensive patterns. However, the findings also indicated some limitations concerning the breadth of PIRS categories, which may overlook the essential nuances of micro‐processes.

**Conclusions:**

While the PIRS has proven its value for understanding clinical micro‐process, its categories may be slightly refined. A proposal for a more granular classification of PIRS categories has finally been provided via a literature analysis.

**Summary**

- The Psychodynamic Intervention Rating Scale (PIRS) is a widely used tool for categorising psychotherapeutic micro‐processes, with applicability across various therapeutic approaches beyond its psychodynamic origins.
- PIRS is particularly effective in assessing therapeutic alliance dynamics and patients' defensive responses, providing clinicians with valuable insights to refine their therapeutic techniques.
- The current categories of PIRS may not fully capture the complexities of therapeutic exchanges, potentially missing important micro‐processes.
- A more refined categorisation of PIRS is suggested to improve its ability to capture the nuances of therapy dynamics.

## 1 Introduction

The focus of psychotherapy research has progressively transitioned from the question of if psychotherapy is effective to a more nuanced investigation of which mechanisms are effective and how they exert these effects (Castonguay 2013) towards the adoption of a micro‐processual perspective (Busch et al. 2009; Falkenström and Larsson 2017; Hayes and Hofmann 2017; Hofmann and Hayes 2019). A micro‐process is defined as a series of point observations throughout the course of psychotherapy encompassing exchanges between therapist and patient that are temporally distinguishable at the level of verbal, paraverbal, behavioural and/or physiological/somatic signals (e.g., Gelo, Pritz, and Rieken 2015; Kleinbub, Talia, and Palmieri 2020). These detailed observations can be utilised for a plurality of purposes, such as testing which mechanisms of change are associated with good outcomes (Falkenström and Larsson 2017; Elliott 2010).

Transcript analysis through ad hoc coding systems stands out as the most widely employed method of conducting micro‐process research. The Psychodynamic Intervention Rating Scale (PIRS; Cooper and Bond 1992) is considered one of the most suitable coding systems for categorising the verbal micro‐process in psychotherapy (Colli, Gagliardini, and Gullo 2022), as it covers a broad range of interventions clustered across 10 categories divided into Non‐interpretive interventions and Interpretive interventions (see Table 1 for details). As formalised by the Expressive–Supportive Intervention Level (ESIL; Despland et al. 2001; see Table 2 for details), PIRS interventions' classification spans across a continuum, ranging from an Expressive polarity, focusing primarily on facilitating patients' confrontation of inner conflicts to promote their resolution by employing incisive solutions, such as offering the interpretations of defence mechanisms to increase their flexibility, up to a Supportive polarity, focusing primarily on supporting patients' symptom reduction and management by, for example, reinforcing defence mechanisms. First conceptualised by Knight (1949, 1952), the expressive–supportive (E–S) continuum has been widely resonant in clinical practice and inspired many psychodynamic psychotherapy guidelines and manuals (e.g., Gabbard 2015; Lingiardi and McWilliams 2020; Luborsky 1984; Sharpless 2019). The PIRS can reliably classify interventions across various therapeutic approaches, for instance, inter‐rater reliability ranges from 91% to 93% for coding in Cognitive Behavioural Therapy (CBT) sessions and from 98% to 100% for psychodynamic and psychoanalytic sessions (Banon et al. 2013). As highlighted in Gumz et al. (2015) seminal review on psychotherapeutic verbal intervention measures, the PIRS enables the analysis of the impact of specific intervention techniques at a micro‐level, contributing to a better understanding of the mechanisms underlying psychotherapeutic change. As a whole, PIRS provided a scientific panorama in which therapists' specific interventions may influence, for instance, the therapeutic alliance, the patient's metacognitive functioning, and the overall level of defences. Despite the widespread use of PIRS in psychotherapy research, a systematic review of its application is currently lacking in the literature. Therefore, the aim of this systematic review is to investigate, according to PRISMA guidelines (Page et al. 2021), the state of the art in the scientific literature of PIRS use, providing a perspective on its application in the last 30 years in developing the understanding of the micro‐process, thus highlighting its strengths and limitations, based on which we will delineate potential future developments in line with needs and trends in psychotherapy process research.

**TABLE 1:** The Psychodynamic Intervention Rating Scale: Summary of categories defined in the manual.

**Therapists' Interpretive interventions**
Defence interpretations (D): identifying, referring to or explaining the motives behind affect‐mitigating processes or shifts in topic content.
Transference interpretations (T): identifying, referring to, inquiring about or explaining the patient's experience of the therapeutic relationship. This coding system requires interventions classified as Defence interpretations (D) and Transference interpretations (T) to be scored on a five‐point scale assessing the depth or completeness of the interventions.
**Therapists' Non‐interpretive interventions**
Acknowledgements (A): confirming receipt of the patient's communication.
Clarifications (CL): summarising the patient's statements without making any interpretations, thereby ensuring accurate understanding.
Questions (Q): regarding the patient's affects, life details, relationships or significant others.
Associations (ASS): reflecting, without making any interpretations, on the patient's previous statements, involving self‐disclosures, factual statements, opinions, answers to questions or explanations.
Reflections (R): concisely conveying the patient's experiences by typically focusing on the expression of an affective state.
Work‐enhancing strategies (WES): emphasising the therapeutic value and rationale of therapy, encouraging the patient to freely express any thoughts.
Support strategies (SS): suggesting, reinforcing or questioning the patient's solutions to different problems.
Contractual arrangements (CA): concerning the timing, duration and specifics of treatment.

**TABLE 2:** Classification of PIRS interventions according to the Expressive–Supportive Intervention Level (ESIL; Despland et al. 2001).

Transference interpretations	7
Defence interpretations (3)	6
Defence interpretations (1)	5
Questions, Clarifications, Work‐enhancing strategies	4
Reflections	3
Support strategies, Contractual arrangements	2
Associations	1

## 2 Methods

### 2.1 Search Strategy

We conducted a systematic review aimed at analysing the contribution of the PIRS in understanding clinical micro‐process, highlighting its strengths and weaknesses. We followed the Preferred Reporting Items for Systematic Reviews and Meta‐Analyses (PRISMA) guidelines (Page et al. 2021). We performed our search from 1992, when the PIRS was first developed by Cooper and Bond (1992), to August 2024. The databases PsycINFO, Scopus, PubMed and Web of Science were searched. Searches were completed separately for each database, utilising the abstract, text and title search fields for all terms. The search term was ‘Psychodynamic Intervention Rating Scale’. In alignment with the PRISMA guidelines, further relevant articles were identified by screening in‐text citations of the selected articles (‘snowballing’, c.f. Greenhalgh and Peacock 2005). As a result, in‐text relevant citations, if peer‐reviewed and indexed, were also considered. After collecting the records and removing duplicates, we applied several eligibility criteria.

### 2.2 Inclusion Criteria

The inclusion criterion that guided the data extraction process consisted of selecting articles that involved the use of the PIRS coding system, at least in part (e.g., used alongside other coding systems). Specifically, we considered only original research studies that explicitly used PIRS as a method for the analysis of verbatim transcripts of psychotherapeutic or counselling sessions of any theoretical orientation. Exclusion criteria included (a) studies that did not explicitly use the PIRS within the research as a coding system (e.g., a citation within the theoretical introduction or the use of the classification without an explicit coding process); (b) papers reporting systematic reviews, single‐case studies, single‐session studies or studies reporting overlapping data with another included study; and (c) non‐peer‐reviewed, non‐indexed or non‐English publications.

### 2.3 Article Selection

**FIGURE 1:**
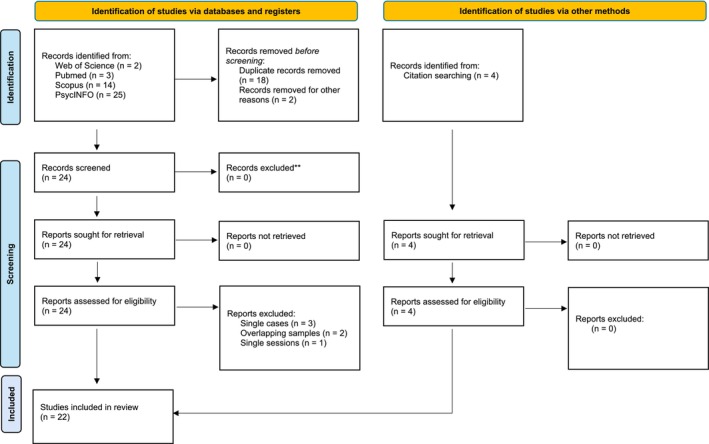
PRISMA flow diagram (Page et al. 2021).

The initial database search yielded 44 papers. The full screening process and reasons for study exclusions are detailed in Figure 1. Among these, 18 articles were removed as duplicated entries, and two reports were excluded for not being pertinent to the research questions. The remaining 24 articles were selected for eligibility assessment. Subsequently, six records had to be excluded for not meeting the inclusion criteria (e.g., single‐case studies). Following the in‐depth research step, four additional studies were identified from citations searching; all 22 included studies are shown in Table 3 in chronological order. These studies are peer‐reviewed articles published in English gathered between 1992 and 2022.

**TABLE 3:** Study syntheses and PIRS contributions.

Authors	Sample (*N*, population information)	Study descriptions	PIRS's contributions to understanding micro‐process
Colli, Gagliardini, and Gullo (2022)	Sixty‐five clinical (DSM‐5) participants (45 F; *M* = 34 years, SD = 4.5) with borderline personality disorder (*n* = 17), narcissistic personality disorder (*n* = 15), schizoid personality disorder (*n* = 6), mood disorders (*n* = 12), anxiety disorders (*n* = 11) or not otherwise specified (*n* = 5).	In both psychodynamic and cognitive treatments, therapists' countertransference responses (i.e., parental, positive, criticised/mistreated, overinvolved and sexualized) (TRQ), completely mediated the relationship between patients' functional levels (DMRS/ODF) and therapists' interventions (PIRS/ESIL).	Expressive Interventions, such as Interpretations and Confrontations, can also be provided by CBT therapists. Expressive Interventions can be elicited by criticised/mistreated therapists' countertransference, while Supportive Interventions can be elicited by positive, overinvolved and sexualised countertransference.
Esposito, Karterud, and Freda (2021)	Seven nonclinical participants (i.e., underachieving university students) (4 F; *M* = 26 years, SD = 6.7).	In narrative group counselling, specific interventions (PIRS and MBT‐G‐AQS) emerged as group members expressed changes in reflective functioning (RFS), which occurred in two ‘shifts’ (i.e., from low RF to ordinary RF and from ordinary RF to high RF). Students' overall reflective functioning (RFS) and their academic achievements (API) showed a significant increase after group counselling.	Empathic Comments by therapists—a category not included in the PIRS—and Clarification Interventions can facilitate an initial change (i.e., the ‘first shift’) in reflective functioning, while Defence interpretations can promote a subsequent change (i.e., the ‘second shift’) in reflective functioning.
Locati et al. (2020)	Forty‐five clinical (SWAP‐200) participants (35 F; *M* = 38.14 years, SD = 11.56) with neuroticism (*n* = 33), schizoid personality disorder (*n* = 1), borderline personality disorder (*n* = 2), histrionic personality disorder (*n* = 1), avoidant personality disorder (*n* = 2), dependent personality disorder (*n* = 2) or obsessive‐compulsive personality disorder (*n* = 4).	In psychodynamic‐oriented therapy, peculiar therapists' interventions (PIRS) were found to be associated with the type of therapeutic alliance, (i.e., ‘positive cycle’, ‘neutral cycle’ and ‘negative cycle’) (CIS). A therapist's expertise was found to be more associated with the type of therapeutic alliance than patients' functioning level (SWAP) and patients' metacognitive function (MAS‐R).	The interventions Transference interpretations, Support strategies and Work‐enhancing strategies can lead to ruptures in the therapeutic alliance (i.e., negative cycle), whereas the interventions Questions, Clarifications and Contractual arrangements can elicit primarily superficial collaborative responses from the patient (i.e., neutral cycle). These two types of association are both directly mediated by a therapist's lack of expertise. Conversely, the greater a therapist's expertise, the more the interventions Acknowledgement and Reflection can foster a positive alliance (i.e., positive cycle).
Bhatia et al. (2019)	Seventeen female clinical (DSM‐IV‐TR) participants (*M* = 24.63 years, SD = 3.63) with anxiety, depressive or personality disorder.	In short‐term dynamic psychotherapy, Defence interpretations (PIRS) were compared across various alliance sessions (Haq‐I) in terms of therapists' verbosity (TVID). In sessions characterised by low alliance (Haq‐I), a significant negative relationship was observed between word length per interpretation and patients' overall defensive functioning (DMRS/ODF).	Defence interpretations characterised by the use of longer words can be negatively associated with a patient's level of defensive functioning if provided in sessions marked by a low‐level therapeutic alliance. Since this finding was not attributable to the length of the intervention itself or to the use of technical terminology, it does not seem to provide clear indications regarding potential variations in a therapist's language.
Locati, Rossi, and Parolin (2019)	Twenty‐four high‐functioning patients (17 F; *M* = 24.06 years, SD = 2.5).	In supportive psychodynamic‐oriented therapy, therapist interventions (PIRS) were found to be associated with the type of therapeutic alliance (i.e., ‘positive cycle’, ‘neutral cycle’ and ‘negative cycle’) (CIS). Patients' metacognitive functioning (MAS‐R) was enhanced by any therapist interventions and played a mediating role in the positive and neutral cycles of the alliance but not in the negative one.	All PIRS interventions seemed to positively impact high‐functioning patients' metacognition. Defence and Transference interpretations as well as Work‐enhancing strategies can lead to ruptures in the therapeutic alliance (i.e., negative cycle). Contractual arrangements and Association interventions can elicit mainly a superficial collaborative response from the patient (i.e., neutral cycle), whereas Acknowledgement and Reflection interventions can foster a positive alliance (i.e., positive cycle).
Esposito, Marano, and Freda (2018)	Two groups of underachieving students with no clinical diagnoses. Group A: five participants (5 F; *M* = 27.86 years, SD = 7.06); Group B: five participants (3 F; *M* = 24.2 years, SD = 5.21).	The frequency of counsellors' interventions (PIRS) of two similar groups undergoing narrative group counselling treatment was evaluated. The depth and frequency of Defence interpretations were greater in the group that appeared to be less dependent on the counsellor.	Questions, Support strategies and Associations are commonly used in counselling treatment. Defence interpretations can be utilised more frequently than Transference interpretations. When group participants demonstrate autonomy from the counsellor (e.g., in turn‐taking), deeper‐level Defence interpretations can be employed.
Petraglia et al. (2018)	Twenty‐two clinical (DSM‐IV‐TR) participants (*M* = 24.36 years, SD = 3.02) with anxiety, depressive or personality disorder.	Lag sequential analysis in short‐term dynamic psychotherapy revealed more frequent patterns of therapist interventions according to alliance in sessions (Haq‐II). Interpretive interventions (PIRS) used with a progressive level of depth occurred in sessions with low therapeutic alliance, whereas sequences made up of Supportive Strategies interventions (PIRS) tended to be more predictable during high‐alliance sessions. Specific interventions responding to particular patients' defences were observed.	Sequences composed of Support strategies can promote high levels of alliance. Mixed interventions sequences, with Interpretive interventions interspersed with Non‐interpretive interventions or solely Interpretive interventions, risk failing to maintain a strong alliance. Specifically, the use of Defence interpretations with increasingly progressive depth can jeopardise alliances.
Bhatia et al. (2016)	Nineteen clinical (DSM‐IV‐TR) participants (7 F; *M* = 24.63 years, SD = 3.46) with anxiety, depressive or personality disorder.	In short‐term dynamic psychotherapy, therapists' Interpretive interventions (PIRS) were found to be primarily directed towards patients' prominent defences (DMRS/ODF) according to their specific characterological/psychopathological profiles, irrespective of the level of the alliance (HAq‐I).	Defence interpretations can be successfully employed in response to patients' typical characterological defences. However, when patients use out‐of‐character defences, therapists can continue to focus on patients' typical characterological defences, which risks either overestimating or underestimating Defence interpretations, thus making them ineffective.
Petraglia et al. (2015)	Thirty‐six clinical (DSM‐IV‐TR) participants (*M* = 23.94 years, SD = 3.93) with anxiety, depressive or personality disorder.	In short‐term dynamic psychotherapy sessions characterised by low alliance, compared to those with high alliance (HAq‐II), a greater frequency and depth of Defence interpretations (PIRS) occurred mainly in association with patients' disavowal defences (projection, denial and rationalisation) (DMRS/ODF).	Defence interpretations are deeper in low‐alliance sessions than in high‐alliance sessions. Given the correlational nature of the data, it remains uncertain whether deeper Defence interpretations weaken the alliance or if a weaker alliance leads to a higher frequency of such interventions. Nonetheless, the way Defence interpretations are structured is associated with the strength of the therapeutic alliance.
Sahli, de Roten, and Despland (2015)	Eleven clinical (DSM‐IV) participants (7 F; *M* = 24.1 years, SD = 3.2) with adaptation disorder (*n* = 6), unipolar depression (*n* = 5) and unspecified Cluster C personality disorders (*n* = 1).	In short‐term dynamic psychotherapy, one rupture and one rupture‐resolution session (HAq‐I) were compared with one control session according to the Index of Interpersonal Accuracy (IA) which is based on the relevance of Interpretive interventions (PIRS) to patients' core conflictual relational theme (CCRT). The results did not reveal significant differences between the sessions, except that higher IA in rupture‐resolution sessions was associated with better therapy outcomes (SCL‐90‐R, BDI, STAI, IIP and OEQ).	When employed in alliance rupture‐resolution sessions, the newly theorised Interpersonal Accuracy index, based on the match between therapists' Interpretive interventions and patients' core conflictual relational themes, can predict both symptom reduction and treatment satisfaction.
Gerostathos et al. (2014)	Seventeen clinical (DSM‐IV) participants with various psychiatric difficulties, including adjustment disorder, major depression, anxiety disorder and Cluster C personality disorders.	In short‐term dynamic psychotherapy, rupture and rupture‐resolution sessions (HAq‐I) were compared according to the Therapist Addressing Defenses (TAD) index, which is based on a selection of therapist Interpretive interventions (PIRS) that directly address patient's defences (DMRS). Rupture sessions and resolution sessions were characterised by fewer and more TADs addressing peculiarly neurotic/obsessive defences, respectively.	Interpretive interventions specifically addressing patients' obsessive neurotic defences, among others (e.g., intermediate defences), differentiated alliance rupture‐resolution sessions from alliance rupture sessions.
Banon et al. (2013)	Eleven clinical (DSM‐IV‐TR; HAMD‐17) participants (8 F; *M* = 37 years) with acute recurrent major depressive episodes; 33 clinical (GSI; BDI) participants (25 F; *M* = 24 years) with a variety of psychiatric disorders (e.g., major depressive episodes, dysthymia, simple phobias, panic disorder, generalised anxiety disorder, adjustment disorder or personality disorder); 35 clinical (DSM‐IV‐TR; GSI) participants (30 F; *M* = 36 years), with occasionally comorbid depressive and anxiety disorders (*n* = 23) and Cluster C personality disorders (*n* = 24); and 17 clinical (DSM‐IV‐TR; HSRS) participants.	Verbatim transcripts of five treatments, conducted with cognitive behavioural, psychodynamic and psychoanalytic approaches, were compared in terms of PIRS, PIRS/ESIL‐1 and PIRS/ESIL‐2* categorisation. A peculiar use of PIRS interventions characterised each approach and the different phases of therapy.	PIRS interventions seemed useful for defining the peculiarity of approaches. Interpretive interventions are more frequently utilised in psychoanalysis and psychodynamic approached compared to CBT, which is characterised by Supportive Interventions. Work‐enhancing strategies, Contractual arrangements and Associations are employed more often in psychoanalysis than in both psychodynamic therapy and CBT.
Romano et al. (2009)	Twenty‐four students (17 female; *M* = 28 years, SD = 10.28) with mild levels of self‐reported concerns, such as relationship difficulties (*n* = 13), self‐esteem problems (*n* = 5), career and academic issues (*n* = 2) and subclinical eating disorders (*n* = 1).	In short‐term counselling, the client's attachment style (ECR) played a moderating role in the relationship between the therapist's attachment style and the therapist's interventions (PIRS) in predicting the frequency of Directive Interventions (i.e., Work‐enhancing strategies, Contractual arrangements and Questions).	Therapists with an avoidant attachment style, when treating patients with the same style, can risk overusing Directive Interventions, such as Work‐enhancing strategies, Contractual arrangements, and Questions, particularly in the early sessions.
Drapeau et al. (2008)	Thirty‐two clinical (DSM‐IV; HAMD‐21; HAMA‐21; SCL‐90) participants (17 F; *M* = 29.97 years, SD = 8.35) with occasionally comorbid mood disorders (*n* = 19), anxiety disorders (*n* = 11) and Cluster C personality disorders (*n* = 12).	In brief psychodynamic interventions, lag sequential analyses revealed relatively stable organised sequences in both therapists' interventions (PIRS/ESIL) and patients' in‐session defensive functioning (DMRS/ODF). No chains of therapist interventions led to a predictable response in patients' defensive functioning. Expressive Interventions were preceded by specific patterns of Supportive Interventions employed by the therapists.	A series (up to four) of Associations and Work‐enhancing strategies, followed by Support strategies, can be used to improve the implementation of Interpretive interventions.
Stigler et al. (2007)	Twenty‐nine clinical (DSM‐IV) participants (16 F; *M* = 29.4 years, SD = 10.1) with (16 F; *M* = 29.4 years, SD = 10.1), with occasionally comorbid mood disorders (*n* = 16), anxiety disorders (*n* = 10), eating disorders (*n* = 2), substance use disorders (*n* = 2), and Cluster C personality disorders (*n* = 9).	In brief psychodynamic investigations, the quality of Interpretive interventions (PIRS) was evaluated with two indices:Accuracy (ACU), which was measured by comparing the patient's CCRT with CCRT inferred from therapist's interpretations, and Conflictuality (CFL), which refers to the number of interpretations involving a conflict between two CCRT components. The sum of the two indices revealed positive associations with an early therapeutic alliance (HAq‐I) and a negative association with patients' interpersonal problems (IIP). A lower ACU was associated with high levels of patients' defensive functioning (DMRS/ODF).	The quality of Interpretive interventions, as defined by accuracy and conflictuality indices, can be associated with the development of a positive early therapeutic alliance. Therapists potentially provide worse Interpretive interventions in terms of accuracy or conflictuality when the patient is more interpersonally controlling. Interpretive interventions tend to be more accurate when provided to patients with lower levels of defensive functioning.
Hersoug, Bøgwald, and Høglend (2005)	Thirty‐nine clinical (DSM‐IV) participants (33 F; *M* = 36.7 years, SD = 8.1) with occasionally comorbid depressive disorders (*n* = 26), anxiety disorders (*n* = 25) and personality disorders (*n* = 25).	In brief psychodynamic psychotherapy, a higher proportion of Interpretive interventions at the end of treatment has been associated with a reduced use of maladaptive defences, but not adaptive ones. Transference interpretations are used more cautiously than Defence interpretations. Supportive Interventions did not significantly impact the development or maintenance of either maladaptive or adaptive defences.	Interpretive interventions can potentially impact the change in maladaptive defence mechanisms after therapy, specifically in terms of reducing the use of acting out, splitting, projective identification and disavowal defences.
Junod et al. (2005)	15 clinical (DSM‐IV) participants (8 F; *M* = 26 years, SD = 6.59) with sometimes comorbid Major Depressive Disorder (*n* = 10), Anxiety Disorder (*n* = 1), Adjustment Disorder (*n* = 3), Cluster C Personality Disorders (*n* = 3), Cluster B Personality Disorders (*n* = 4), and Cluster A Personality Disorder (*n* = 1).	In brief psychodynamic interventions, TAD accuracy (i.e., the comparison between the effective patient's defence and the defence addressed by the therapist's Interpretive interventions) was associated with specific types of therapeutic alliance (HAq‐I).	When the therapeutic alliance is low, therapists may be less accurate in terms of interpreting patients' defence as more immature. When the alliance is high, although Defence interpretations tend to be more accurate with respect to patients' specific defences, therapists may sometimes interpret these defences as more mature than they truly are (they tend to neuroticize the patient).
Terraz et al. (2004)	Twenty clinical participants (13 F; *M* = 30.40 years, SD = 12.49). Therapist‐patient dyads were assigned to three groups:a high and stable alliance profile (HSA), a low and stable alliance profile (LSA) and an improving alliance profile (IA).	In brief psychodynamic investigations, lag sequential analysis was employed to determine the relationship between intervention patterns (PIRS) and the therapeutic alliance (HAq‐I). Mixed sequences of interventions were positively associated with a stably high or improving therapeutic alliance over the course of treatment. Conversely, a stably low alliance was observed when sequences consisted solely of Interpretive interventions.	Both sequences of Questions, Associations, Clarifications and Therapeutic Frame Interventions (i.e., Contractual arrangements and Work‐enhancing strategies) and mixed sequences of Interpretive interventions, Reflections and Support strategies can lead to a stably high or improving alliances. Conversely, single sequences of Interpretive or Supportive Interventions can maintain a stably low alliance relative to the use of mixed sequences.
Despland et al. (2001)	Twelve clinical (DSM‐IV) participants (11 F; *M* = 28.5 years, SD = 12.5) diagnosed with anxiety disorders, depressive disorders or personality disorders.	In brief psychodynamic investigations, the correspondence between intervention levels (PIRS/ESIL) and patients' overall defensive functioning (DMRS/ODF)—namely, Adjustment Ratio (AR)—in the first session was able to predict the quality of the alliance (HAq‐I) in the three subsequent sessions. AR differed significantly among the group characterised by a stably high alliance, which showed a higher PIRS/ESIL score in the first session, with respect to the groups characterised by an improving alliance and a stably low alliance. The latter had the lowest mean AR across all the sessions.	Regardless of patients' peculiar level of defensive functioning, when therapeutic interventions are specifically adjusted to patients' overall defensive functioning, a positive therapeutic alliance can occur. Interpretive/Expressive Interventions can ensure a high and stable therapeutic alliance from the first session. Conversely, a higher proportion of Supportive Interventions compared to Interpretive interventions can result in a consistently low alliance.
Banon, Evan‐Grenier, and Bond (2001)	Seven clinical (DSM‐III‐R) participants with one or more personality disorder diagnoses and/or a current mood disorder or anxiety disorder diagnosis.	In long‐term dynamic psychotherapy involving patients with personality disorders, early Transference interpretations (PIRS) were associated with increased defensiveness, even within a strong alliance (CALPAS). Sequences of interventions that included early Transference interpretations combined with Defence interpretations or Non‐interpretive interventions were associated with better therapeutic alliances without increasing defensiveness or resistance.	The use of Transference interpretations alone can jeopardise the alliance. However, within the context of a solid early alliance and when therapists also provide Non‐interpretive interventions and Defence interpretations, early Transference interpretations can lead to enhanced therapeutic work.
Milbrath et al. (1999)	Twenty clinical (SCL‐90; BPRS; GAS) participants matched for gender (all female) and age (*M* = 40.2 years) exhibiting bereavement ranging from normal to pathological grief reactions.	The PIRS was assessed for reliability and construct validity, with positive results in comparison to the SCL‐90, BPRS and GAS. A sequential analysis revealed that therapists adjusted their intervention styles based on patients' levels of distress and functioning (SCL‐90, BPRS and GAS). Specific patterns of both Interpretive and Supportive Interventions were predictive of positive therapy outcomes.	Defence and Transference interpretations are confirmed to be effective in promoting the processes of meaning‐making of experience and emotional patient disclosures. Questions, Associations, Clarifications and Contractual arrangements tend to lead patients to provide factual information rather than emotional content. Support strategies and Work‐enhancing strategies can be employed with patients exhibiting higher levels of symptoms and distress, whereas Questions can be used when patients self‐report greater depression. Reflections, Defence interpretations and Support strategies can lead to reductions of symptoms and improved levels of functioning after therapy.
Bond, Banon, and Grenier (1998)	Five clinical (DSM‐III‐R) participants (5 F) diagnosed with borderline personality disorder (*n* = 4), dysthymic disorder and narcissistic personality disorder (*n* = 1).	In long‐term dynamic psychotherapy involving patients with personality disorders, Transference interpretations (PIRS) were influenced by the state of the therapeutic alliance (CALPAS), phase of therapy and timing within the session. Defence interpretations consistently contributed to enhancing the therapeutic alliance. Non‐interpretive interventions addressed alliance ruptures and were used in preparation for subsequent Interpretive interventions.	In the early phase of therapy, Transference interpretations are high‐risk interventions that can lead to a deterioration in the therapeutic alliance if the alliance is weak; however, they can foster enhanced therapeutic work if the alliance is solid. Defence interpretations and Non‐interpretive interventions can improve therapeutic work without increasing defensiveness, regardless of the state of the therapeutic alliance.

Abbreviations: API, Academic Performance Inventory; AR, Adjustment Ratio; BDI, Beck Depression Inventory (Beck and Beamesderfer 1974; Beck and Steer 1993); BPRS, Brief Psychiatric Rating Scale (Overall and Gorham 1962); CALPAS, California Psychotherapy Alliance Scale (Marmar et al. 1989); CCRT, Core Conflictual Relationship Theme (Luborsky 1990); CIS, Collaborative Interactions Scale (Colli and Lingiardi 2009); DMRS, Defense Mechanism Rating Scale (Perry 1990, 2001); DS, Dynamic Scale (Høglend et al. 2000); DSM‐III‐R, Diagnostic and Statistical Manual of Mental Disorders, 3rd ed., rev. (American Psychiatric Association 1987); DSM‐IV, Diagnostic and Statistical Manual of Mental Disorders, 4th ed. (American Psychiatric Association 1994); DSM‐IV‐TR, Diagnostic and Statistical Manual of Mental Disorders, 4th ed., text rev. (American Psychiatric Association 2000); DSM‐5, Diagnostic and Statistical Manual of Mental Disorders, 5th ed. (American Psychiatric Association 2013); DTI, Dynamic Tasks for Interviewing (Perry et al. 2005); ESIL, Expressive–Supportive Intervention Level (Despland et al. 2001; Hersoug, Bøwald, and Høglend 2003); EXP, Experiencing Scale (Klein et al. 1961); GAS, Global Assessment Scale (Endicott et al. 1976); GSI, Global Severity Index (Derogatis et al. 1973); HAMA‐21, Hamilton Anxiety Scale (Hamilton 1959); HAMD‐17, Hamilton Depression Rating Scale 17‐item version (Hamilton 1960); HAMD‐21, Hamilton Depression Rating Scale 21‐item version (Hamilton 1960); HAq‐I, Helping Alliance Questionnaire‐I (Alexander and Luborsky 1986); HAq‐II, Helping Alliance Questionnaire‐II (Luborsky et al. 1996); HSRS, Health Sickness Rating Scale (Luborsky 1962); I‐DIA, Interviewer's Dynamic Interview Adequacy (Fowler and Perry 2006); IIP, Inventory of Interpersonal Problems (Horowitz et al. 1988); MAS‐R, Metacognition Assessment Scale‐Revised (Carcione et al. 2010); MBT‐G‐AQS, Mentalization‐Based Treatment for Groups Adherence and Quality Scale (Karterud 2015); ODF, Overall Defensive Functioning; O‐DIA, Overall Dynamic Interview Adequacy (Fowler and Perry 2006); OEQ, Outcome Evaluation Questionnaire (Sahli, de Roten, and Despland 2015); PIRS, Psychodynamic Intervention Rating Scales (Cooper and Bond 1992); RF, Reflective Function; RFS, Reflective Functioning Scale (Fonagy et al. 1998); SCL‐90, Symptom Check‐List (Derogatis et al. 1973); SCL‐90–R, Symptom Check‐List (Derogatis 1994); STAI, State–Trait Anxiety Inventory (Spielberger and Gorsuch 1966; Spielberger et al. 1970); S‐DIA, Subject Dynamic Interview Adequacy (Fowler and Perry 2006); SWAP‐200, Shedler‐Westen Assessment Procedure (Westen and Shedler 1999); TAD, Therapist Addressing Defense; TRQ, Therapist Response Questionnaire (Betan et al. 2005; Tanzilli et al. 2016); WAI, Working Alliance Inventory (Horvath and Greenberg 1994).

### 2.4 Data Extraction

The following information were extracted for each included study: authors, year of publication, sample size, population details (clinical/non‐clinical sample, any diagnoses for the clinical group), study description and key findings related to the PIRS. Specifically, three authors (V.C.P., D.R. and V.C.) independently reviewed the titles, abstracts and keywords of all retrieved records and discussed inconsistencies until consensus was achieved regarding the eligibility of articles based on the established inclusion criteria. The first author (A.P.) was consulted before any final decisions were made. The same procedure was completed in the inclusion for full‐text articles.

### 2.5 Data Synthesis

The selected articles were detected. Next, the specific contributions arising from the use of PIRS were identified. Three authors (V.C.P., D.R. and V.C.) first identified the main results of the included articles, compared their findings and synthesised and summarised the contributions. In both steps, inconsistencies were resolved by consulting the first author (A.P.). These study characteristics were compiled into a table to provide an overview of the included studies (Figure 1).

## 3 Results

All 22 included studies referred to specific therapeutic approaches. The PIRS was utilised in six studies of short‐term dynamic psychotherapy (STDP; Gilliéron 1997), four studies of dynamic psychotherapy and two studies on long‐term psychodynamic therapy, one study on brief dynamic psychotherapy and three studies on brief psychodynamic investigation (Gilliéron 1989). Additionally, the PIRS was employed in two studies of Brief Psychodynamic Intervention (Gilliéron 1994, 1997) and in one study of long‐term psychoanalytic treatments. Furthermore, the PIRS was employed in CBT in two studies, while in four studies it was applied in studies involving group counselling (*n* = 3) and brief therapy for severe grief reactions (*n* = 1). Of the selected studies, 19 were conducted on a clinical sample, while three involved a nonclinical population (e.g., students with moderate academic difficulties). These studies encompassed a total of 574 clinical (*n* = 531) and nonclinical (*n* = 43) subjects. Studies that specified the number of patients with specific diagnoses predominantly comprised patients with personality disorders (*n* = 134), followed by anxiety disorders (*n* = 81) and mood disorders (*n* = 122). The other studies did not specify the number of patients with specific diagnoses.

The relationship between PIRS categories and the nature and quality of the therapeutic alliance has been examined in 13 studies (Banon, Evan‐Grenier, and Bond 2001; Bhatia et al. 2019; Bond, Banon, and Grenier 1998; Despland et al. 2001; Gerostathos et al. 2014; Junod et al. 2005; Locati, Rossi, and Parolin 2019; Locati et al. 2020; Petraglia et al. 2015, 2018; Sahli, de Roten, and Despland 2015; Stigler et al. 2007; Terraz et al. 2004), revealing distinct patterns for each type of alliance (Despland et al. 2001; Junod et al. 2005; Locati, Rossi, and Parolin 2019; Locati et al. 2020; Petraglia et al. 2015, 2018; Terraz et al. 2004) accordingly to the specific alliance measures employed by the authors. Expressive Interventions have been associated with ruptures or deterioration in therapeutic alliances (Banon, Evan‐Grenier, and Bond 2001; Bond, Banon, and Grenier 1998; Locati, Rossi, and Parolin 2019; Locati et al. 2020). Conversely, Supportive Interventions have generally been associated with a positive alliance (Bond, Banon, and Grenier 1998; Locati, Rossi, and Parolin 2019; Locati et al. 2020; Petraglia et al. 2018), with certain exceptions (e.g., eliciting only a superficial collaborative response in the patient) (Locati, Rossi, and Parolin 2019; Locati et al. 2020).

PIRS categories have also been associated with the patient's defence functioning in nine studies (Bhatia et al. 2016, 2019; Colli, Gagliardini, and Gullo 2022; Despland et al. 2001; Drapeau et al. 2008; Gerostathos et al. 2014; Hersoug, Bøgwald, and Høglend 2005; Petraglia et al. 2015; Stigler et al. 2007), six of which are also considered to be associated with therapeutic alliance (Bhatia et al. 2016, 2019; Despland et al. 2001; Gerostathos et al. 2014; Petraglia et al. 2015; Stigler et al. 2007) and five of which have concerned Defence interpretations (Bhatia et al. 2016, 2019; Gerostathos et al. 2014; Junod et al. 2005; Petraglia et al. 2015). Defence interpretations primarily target patients' characterological and psychopathological defences (Bhatia et al. 2016), and the PIRS's contributions to associating these interventions with patients' level of functioning remains ambiguous (Hersoug, Bøgwald, and Høglend 2005; Petraglia et al. 2015).

In four studies, the PIRS was associated with the therapeutic approach employed by therapists, revealing peculiarities in the implementation of specific intervention patterns (Banon et al. 2013; Esposito, Marano, and Freda 2018; Esposito, Karterud, and Freda 2021; Romano et al. 2009). Banon et al. (2013) observed that CBT employs more Supportive Interventions (according to ESIL) compared to dynamic/psychoanalytic therapies, which favour Expressive Interventions (e.g., Transference and Defence interpretations), whereas Work‐enhancing strategies, Contractual arrangements and Associations are notably more frequently employed in psychoanalysis than in other approaches. Further differences can also be observed within Supportive Interventions in terms of their frequency of use (Esposito, Marano, and Freda 2018).

Interpretive interventions are more common in the middle and later phases of treatment than in the early phase (Banon et al. 2013), and specific sequences of interventions are employed to prepare for them (Banon, Evan‐Grenier, and Bond 2001; Bond, Banon, and Grenier 1998; Drapeau et al. 2008). The accuracy of Interpretive interventions has been directly associated with therapeutic outcomes (Sahli, de Roten, and Despland 2015).

Additionally, several studies have investigated the relationship between PIRS interventions and therapists' expertise (Locati et al. 2020) with patients' levels of symptomatology (Milbrath et al. 1999). Empathic Comments (see limitations paragraph above), Clarifications and Defence interpretations have been found to promote shifts in patients' reflective functioning (Esposito, Karterud, and Freda 2021). Work‐enhancing strategies, Contractual arrangements and Questions (‘directive interventions’) are provided by therapists with avoidant attachment styles when clients exhibit high levels of attachment avoidance (Romano et al. 2009). Therapist's countertransference has been explored in only one study, revealing that Expressive Interventions have been linked to criticised or mistreated countertransference, whereas Supportive Interventions have been linked to positive, overinvolved and sexualized ones (Colli, Gagliardini, and Gullo 2022).

Of the 22 studies analysed, seven studies (Banon et al. 2013; Esposito, Karterud, and Freda 2021; Gerostathos et al. 2014; Milbrath et al. 1999; Romano et al. 2009; Sahli, de Roten, and Despland 2015; Terraz et al. 2004) identified some limitations regarding the PIRS categories, specifically concerning the broadness of the interventions that belong to the Supportive polarity.

The study details, including study syntheses and PIRS contributions, are specified in detail in Table 3. The studies examined have been presented in chronological order, beginning with the most recent, to provide a progression of the contributions to the field.

## 4 Discussion

The main objective of this study was to conduct a systematic review under PRISMA guidelines to examine the current state of the art on the use of the PIRS (Cooper and Bond 1992) in psychotherapy verbatim transcripts—from its validation to the present. We focused on how the PIRS has contributed to advancing the understanding of psychotherapeutic micro‐processes by examining the association of specific categories or broader categories (e.g., Supportive vs. Expressive polarities) with distinct psychotherapy constructs, objectives and factors. Additionally, we provided a brief review of the limitations of the PIRS, as explicitly stated by the authors or clearly inferred from the reviewed contributions and discussed possible enhancements.

### 4.1 PIRS Interpretive/Expressive Interventions

Transference and Defence interpretations are the most investigated PIRS categories in the reviewed studies: of the 22 included empirical studies, eight focused exclusively on Interpretive interventions (Banon, Evan‐Grenier, and Bond 2001; Bhatia et al. 2016, 2019; Gerostathos et al. 2014; Junod et al. 2005; Petraglia et al. 2015; Sahli, de Roten, and Despland 2015; Stigler et al. 2007); among these, four studies concentrated selectively on Defence interpretations (Bhatia et al. 2016, 2019; Junod et al. 2005; Petraglia et al. 2015). In detail, Defence interpretations were found to be employed more frequently than Transference interpretations, which therapists were more hesitant to provide (Hersoug, Bøgwald, and Høglend 2005). Expressive/Interpretive interventions have been found to be predominantly utilised in the latter half of therapy (Banon et al. 2013) in line with the theoretical assumption of delaying interpretations until a holding environment renders them accessible to the patient's awareness (Gabbard 2006), thereby avoiding the risk of premature treatment termination (Migone 1982). Notably, when these interventions are less employed during both the early and middle phases of therapy, they appear to be more significantly influenced by the therapist's experience and specific personality characteristics (Hersoug, Bøwald, and Høglend 2003). Both Transference and Defence interpretations are more prevalent in dynamic and psychoanalytic approaches compared to CBT (Banon et al. 2013) and counselling therapy (Esposito, Marano, and Freda 2018; Esposito, Karterud, and Freda 2021). This observation aligns with their established significance in the psychodynamic approach (e.g., Antichi, Giannini, and Loscalzo 2022; Shedler 2010). Many authors over nearly a century have indeed considered interpretations a central feature of psychodynamic psychotherapy (Gill and Hoffman 1982; Kernberg 2016; Strachey 1934) due to their role in uncovering unconscious conflicts and desires, facilitating patient insight (Gabbard 2018; Gabbard and Westen 2003), which in turn mediates change (Johansson et al. 2010). However, it is worth noting that Defence interpretations were nevertheless identified in CBT therapies as well (Banon et al. 2013; Colli, Gagliardini, and Gullo 2022), where Expressive Interventions, although limited, increased during the later sessions of therapy (Banon et al. 2013). The use of Expressive Interventions in CBT therapies can be attributed to many factors, which are not solely linked to therapists' theoretical eclecticism but also arise from the polysemy of constructs, that is, an issue that characterises all psychotherapy schools from various perspectives (Palmieri et al. 2022). For instance, in CBT, interventions aimed at confronting or interpreting patients' relational schemes or exploring thoughts and beliefs can, in some cases, be categorised as low‐level Interpretive interventions (Banon et al. 2013; Colli, Gagliardini, and Gullo 2022). The occurrence of Defence interpretations has also been observed, as described by Esposito, Marano, and Freda (2018) and Esposito, Karterud, and Freda (2021), in treatment traditionally associated with Supportive Interventions, such as brief counselling (White and Kelly 2010). Notably, interpretations emerged when clients were perceived by therapists as being less dependent on them (Esposito, Marano, and Freda 2018), and, interestingly, their use led to the occurrence of the deepest shift in patients' reflective functioning, referred to as the ‘second shift’ (Esposito, Karterud, and Freda 2021). Previous studies have confirmed that Expressive Interventions conducted in non‐dynamic therapies can play a significant positive role across various treatment modalities (Jennissen et al. 2018; Shedler 2010). In supportive approaches, such as brief counselling, authors have indeed pointed out that interpretations are one of several distinct skills that may be used to facilitate exploration and effect change (e.g., Ivey and Authier 1978) as, for instance, they convey information not within the client's immediate awareness and, in doing so, promote client experiencing (Gendlin 1968). Interpretive interventions have been broadly studied in relation to their role in fostering or potentially jeopardising the therapeutic alliance. As predicted by other studies, Interpretive interventions are utilised in sessions characterised by a high and stable therapeutic alliance (Despland et al. 2001), whereas their reduced use has been observed in sessions marked by ruptures (Gerostathos et al. 2014). On the other hand, some authors have also demonstrated that Interpretive interventions, when provided inappropriately by therapists, can contribute to deteriorations or ruptures in the therapeutic alliance (Banon, Evan‐Grenier, and Bond 2001; Bond, Banon, and Grenier 1998; Locati, Rossi, and Parolin 2019; Locati et al. 2020; Petraglia et al. 2015; Terraz et al. 2004). Such technical misuse by therapists, whether in the context of supportive therapy or due to a lack of clinical experience (Locati, Rossi, and Parolin 2019; Locati et al. 2020), can be the causes of these findings. In particular, when Transference interpretations are utilised in the early phases of therapy, particularly with patients diagnosed with borderline and narcissistic personality disorders, the risk of compromising alliance‐building has been found to be significant (Banon, Evan‐Grenier, and Bond 2001; Bond, Banon, and Grenier 1998). Notably, if Transference interpretations are followed by Defence interpretations, the therapeutic alliance is maintained even during the early phases of therapy (Banon, Evan‐Grenier, and Bond 2001). The contribution of PIRS in understanding alliance‐related micro‐process has also been jointly investigated with patients' defensive functioning. Bhatia et al. (2016) found that Defence interpretations primarily address patients' typical defence patterns, and in sessions marked by a high therapeutic alliance, the level of such interpretations closely corresponds with the predominant level of the patients' defence mechanisms (Junod et al. 2005). However, Bhatia et al. (2016) showed that therapists who focus solely on interpreting typical defences risk neglecting atypical ones, thereby failing to fully account for micro‐changes in the patient's defensive style, which may lead to potential overestimations or underestimations of the defence mechanisms employed by the patient. Petraglia et al. (2015) observed a greater frequency and depth of Defence interpretations in sessions characterised by a low therapeutic alliance, primarily associated with patients' disavowal defences, such as projection, denial and rationalisation. Since the data are correlational, it is not possible to determine whether a therapist's more frequent and deeper interpretations result in a weaker alliance or whether difficulties in the therapeutic alliance lead therapists to provide these types of interpretations to cope with the therapeutic situation. Focusing exclusively on defensive functioning, Interpretive interventions have been found to foster changes in the patient's maladaptive defences, for example, acting out, splitting, projective identification and disavowal (Hersoug, Bøgwald, and Høglend 2005). However, in a study by Colli, Gagliardini, and Gullo (2022) involving 65 patients who primarily had borderline and narcissistic personality disorders, the use of PIRS provided evidence that both Defence interpretations and Transference interpretations were riskily adopted when therapists experienced Criticized Countertransference. This result suggests that when therapists feel criticised and experience hostility towards their patients, they may react by masking these feelings and expressing them through asymmetrical and distancing interventions, used as a form of intellectualisation. This defence used by therapists is not well‐suited to the clinical moment and can potentially lead to providing less protection to the patient (Colli, Gagliardini, and Gullo 2022), in line with the results by Petraglia et al. (2015) mentioned above. A previous empirical study by Colli et al. (2014) illustrates that borderline and narcissistic patients tend to elicit countertransference feelings of helplessness/inadequacy, overwhelm/disorganisation and either overinvolvement or disengagement from the therapist.

### 4.2 PIRS Non‐Interpretive/Supportive Interventions

As in the case of Expressive/Interpretive Interventions, differences between psychotherapy models have also been observed in Non‐interpretive/Supportive Interventions, confirming the efficacy of PIRS in capturing the nature of therapeutic approaches across interventions. Supportive Interventions occur more frequently in CBT than in psychodynamic therapies, which, in turn, use these interventions more frequently than psychoanalytic therapy. In psychoanalysis, Supportive Interventions are more often adopted at the beginning of therapy, consistent with the necessity of setting the therapeutic frame and explaining the value and rationale of the therapeutic process (e.g., Work‐enhancing strategies and Contractual arrangements), and they become infrequent in the second half of therapy (Banon et al. 2013). Notably, Clarifications and Reflections, which are contiguous in the continuum delineated by Despland et al. (2001), remain stable across all the compared approaches. This can be explained by considering the placement of these interventions with respect to the two polarities of Supportive and Expressive. As for specific patients, Milbrath et al. (1999) found that therapists are prone to adopt Support strategies and Work‐enhancing strategies with subjects presenting higher levels of symptoms and signs of distress, while Questions are mostly used when subjects self‐report higher levels of depression, consistent with the idea that patients with severe symptomatology or low functioning levels are rarely capable of engaging with high‐complexity interventions such as Interpretative ones (McWilliams 2006). Within the context of counselling, Esposito, Marano, and Freda (2018) found a predominant use of Questions, Support strategies and Associations, confirming the distinctive feature of counselling in supporting clients by enhancing their competencies facilitating their re‐authoring of their experiences (Freedman and Combs 1996). Moreover, Work‐enhancing strategies, jointly with Contractual arrangements, can be used to define the rules of the therapeutic setting and were indeed classified as ‘Therapeutic Frame Interventions’ due to their specific use in addressing the therapeutic frame, according to Terraz et al. (2004). Supportive Interventions have been studied in relation to the therapeutic alliance. In two studies by Locati, Rossi, and Parolin (2019); Locati et al. (2020), Supportive Interventions at the beginning of the E–S continuum, namely, Questions, Clarifications, Associations and Contractual arrangements, were found to lead to the formation of a neutral alliance. This can be explained by the fact that these interventions may encourage collaboration only at a superficial level as they address content that lacks emotional nuance. Milbrath et al. (1999) similarly found that these same therapeutic interventions were followed by patients conveying facts rather than emotions. Other Supportive Interventions appear to be associated with the establishment and maintenance of a strong therapeutic alliance (Bond, Banon, and Grenier 1998; Petraglia et al. 2018), independent from levels of patient functioning (Banon, Evan‐Grenier, and Bond 2001; Bond, Banon, and Grenier 1998; Locati, Rossi, and Parolin 2019; Locati et al. 2020; Terraz et al. 2004). This is consistent with findings from several authors regarding the role of Non‐Interpretive/Supportive Interventions in enhancing collaborative processes and creating and maintaining a holding environment that encourages patients to express their experiences and feelings (Ackerman and Hilsenroth 2003; Gabbard et al. 1994; Horwitz et al. 1996; Leibovich et al. 2018; Skean 2005). It is interesting to note that among these interventions, contrary to what is stated in the E–S continuum model by Gabbard (2015) and although the common clinical idea is that such interventions are empathetic in nature (Jungbluth and Shirk 2009; Karver et al. 2008), the role of empathy has not been explicitly reported in any Non‐Interpretive PIRS categories. In this vein, Esposito, Karterud, and Freda (2021) introduced a new category among Supportive Interventions, specifically called Empathic Comments, which contribute to facilitating a ‘first shift’ in therapy, that is, a change from a lower to a higher level of reflective functioning. Colli, Gagliardini, and Gullo (2022) found that Supportive Interventions are applied in greater frequency not only in cases of positive countertransference, but also in instances of overinvolved and sexualised countertransference. The authors explain these results by stating that therapists who experience an overinvolved relationship with their patients are less inclined to provide interventions such as Confrontations and Interpretive interventions, which may be more difficult for patients to accept, opting instead for interventions that are more ‘protective’ of their relationship and do not threaten the ‘honeymoon’ phase of the positive therapeutic alliance. Furthermore, Romano et al. (2009) observed that when both the patient and the therapist exhibit an avoidant attachment style, the therapist tends to refrain from implementing Interpretive interventions, colluding with clients to avoid negative emotions by opting for directive Non‐interpretive interventions—particularly Work‐enhancing strategies, Contractual arrangements and Questions—that focus on the structure of therapy rather than on the expression of emotion or exploration of defences.

### 4.3 Sequence Analysis of PIRS Interventions

Some of the examined studies have identified sequences of interventions associated with specific clinical objectives. Terraz et al. (2004) identified, through lag sequential analysis, some intervention strings leading to three types of therapeutic alliance (i.e., high and stable, low and stable and improving alliance). The key distinction between the different alliance conditions seems to lie in the heterogeneity versus homogeneity of the intervention sequences. Sequences of Questions, Associations, Clarifications and Therapeutic Frame Interventions (i.e., Contractual arrangements and Work‐enhancing strategies) a chain with mixed sequences of Interpretive interventions, Reflections and Support strategies seem to lead to a stably high or improving alliance condition. Strings characterised by the presence of interventions that involved either entirely Interpretive interventions or entirely Non‐interpretive interventions seem to lead, on the other hand, to a stably low alliance. Such a result is also in line with a previous study (Drapeau et al. 2018), which, although based only on the comparison of two single cases—and therefore not included in this systematic review—found that, in successful therapeutic cases, therapists adopted Defence interpretations alongside Questions, whereas unsuccessful cases involved a sequence of purely Interpretive interventions. Conversely, Petraglia et al. (2018), despite the similarity of their sample respect to Terraz et al. (2004) one (i.e., University of Lausanne students diagnosed with anxiety and depression), observed that high‐alliance sessions were predominantly characterised by sequences of exclusively Supportive Interventions, while in low‐alliance sessions, both mixed sequences of Interpretive interventions interspersed with Non‐interpretive interventions or solely Interpretive interventions were found. The discrepancy between the studies by Terraz et al. (2004) and Petraglia et al. (2018) may be attributed to the former's focus on overall alliance scores rather than an analysis of the cycles of low‐alliance and high‐alliance sessions.

In line with scientific and clinical literature (Horowitz 2013; Perry and Bond 2005), Supportive Interventions have been described by several authors in our review as a tool to prepare patients for the receptiveness of subsequent Interpretive interventions (Banon, Evan‐Grenier, and Bond 2001; Bond, Banon, and Grenier 1998; Esposito, Karterud, and Freda 2021; Drapeau et al. 2008). In detail, intervention patterns frequently adopted to anticipate Interpretive interventions consisted of a series (up to four) of Non‐interpretive interventions (i.e., Associations followed by Work‐enhancing strategies) followed by Support strategies (Drapeau et al. 2008). Esposito, Karterud, and Freda (2021) found that when Clarifications were successfully adopted to precede subsequent Defence interpretations, they improved clients' reflective functioning. The effectiveness of the mixed use of Expressive Interventions and Supportive Interventions, such as alternating Support strategies and Reflections with Defence interpretations, was demonstrated in a study by Milbrath et al. (1999) in terms of a reduction in signs of distress and self‐reported symptoms and a higher post‐therapy level of functioning.

### 4.4 Indices Derived From PIRS Categories

Some studies have also proposed indices for measuring the clinical process, including the categories of the PIRS. Among these, the Therapist Addressing Defense (TAD), introduced by Junod et al. (2005), concerns therapist‐employed Interpretive interventions that address defences. The authors found that in sessions with a TAD index categorised as accurate, in terms of high correspondence between Defence Interpretation in response to the specific defence employed by patient's, a stronger therapeutic alliance occurs. In a subsequent study by Gerostathos et al. (2014), the use of TAD demonstrated that in sessions characterised by ruptures, therapists tend to focus less on obsessional and neurotic defences (e.g., intellectualisation, reaction formation, repression or displacement) compared to resolution sessions. This result is in line with the idea that obsessional, neurotic defences play a peculiar role in the regulation of the therapeutic relationship (Foreman and Marmar 1985) and avoiding their interpretation can be detrimental to maintaining the alliance. Moreover, three further indices involving the convergence between PIRS Interpretive interventions and the patient's Core Conflictual Relationship Theme (CCRT) were detected. The first one, Interpersonal Accuracy (IA; Sahli, de Roten, and Despland 2015), refers to the level of adherence of the Interpretive interventions to the patient's CCRT. The second, the Accuracy of Interpretations index (ACU; Stigler et al. 2007), compares the patient's CCRT with the therapist's CCRT interpretations to obtain a rating based on identically organised material on both sides. Finally, the Conflictuality of Interpretations index (CFL; Stigler et al. 2007) is defined as the relative number of Interpretive interventions containing an opposition between two components of the CCRT structure (i.e., Wish‐Response from Others and Response from the Self). Similar to TAD, these indices revealed that the quality of Interpretive interventions and their micro‐processual effectiveness in the therapeutic process depend on their congruence with the patient's problem focus, that is, on their being accurate and containing conflictual elements (Sahli, de Roten, and Despland 2015; Stigler et al. 2007). In this vein, Interpretive interventions linked to patients' CCRT have been found to be predictive of positive therapy outcomes (Sahli, de Roten, and Despland 2015) and the development of positive early therapeutic alliance (Stigler et al. 2007), thus indicating the crucial role of providing Interpretive interventions closely aligned with the patient's experiential meanings (Crits‐Christoph and Gibbons 2001; Safran, Muran, and Eubanks‐Carter 2011) to achieve better therapeutic outcomes (Høglend et al. 2011). However, patient characteristics, such as lower levels of defensive functioning and interpersonal problems (e.g., controlling behaviours), can interfere with the quality of the therapist's Interpretive interventions (Stigler et al. 2007). Finally, the Adjustment Ratio index (AR; Despland et al. 2001) evaluates the adaptation of the therapist's interventions (ESIL) to a given patient's level of defensive functioning (ODF), thus revealing that this adjustment of the therapist's interventions is predictive of good therapeutic alliance, in line with the theoretical assumption that therapists should consider patients' level of functioning and ego strength to achieve desirable outcomes (Laaksonen et al. 2012).

### 4.5 PIRS Limitations and Proposal for Future Perspectives

Some potential improvements could be introduced in light of the issues identified in our review regarding PIRS categorisation, as noted by many authors themselves. Specifically, the broadness of the categories may have compromised the accuracy of the measurement in capturing the underlying clinical process (Banon et al. 2013; Esposito, Karterud, and Freda 2021; Gerostathos et al. 2014; Romano et al. 2009; Sahli, de Roten, and Despland 2015; Terraz et al. 2004), beginning with the initial study on the reliability and construct validity of PIRS (Milbrath et al. 1999). These concerns primarily pertain to the interventions associated with Supportive polarity. To address these limitations, more detailed classifications of interventions within the existing categorisation have been proposed in the examined studies (Banon et al. 2013; Esposito, Karterud, and Freda 2021; Gerostathos et al. 2014; Romano et al. 2009; Sahli, de Roten, and Despland 2015; Terraz et al. 2004). From a micro‐processual analysis perspective, the limitations of PIRS categories' definitions risk obscuring the technical nuances of therapeutic intentions. A more precise definition could also enhance our understanding of the partially inconsistent findings that emerged from our review. In light of the emerging issues, we propose a further refinement of Non‐interpretive categories within the PIRS coding system, namely, Questions (Q), Support strategies (SS), Work‐enhancing strategies (WES), Clarification (CL) and Associations (ASS) interventions, strictly based on the definitions provided in the manual. In detail, the elements of each category could be specified with an alphanumeric code. As for Questions, a proposed further classification involves labelling Q1 for interventions posed by the therapist to obtain information about the patient's life from a factual perspective, and Q2 for those concerning the patient's inner dynamics related to relationships and affects. This greater level of detail could help emphasise what characterises a pure Question and aids in distinguishing it from a component of an interpretive intervention, a concern raised by Milbrath et al. (1999). Support strategies could be categorised into SS1, which indicates interventions where the therapist provides suggestions about the patient's solutions to various problems; SS2, which marks interventions that primarily cognitively validate/reinforce the patient's previous experiences/strategies; and SS3, which primarily empathetically validate/reinforce the patient's previous experiences/strategies. This further refinement could reduce the risk of apparent conceptual overlap noted by Terraz et al. (2004). Moreover, in this vein, it could contribute to bridging the lack of reference to empathic interventions in the PIRS, which is crucial for patient–therapist attunement (Kernberg et al. 2008), as expressed in Esposito, Karterud, and Freda' (2021) contribution. Work‐enhancing strategies (WES) could be further refined into WES1 to delineate the therapist's interventions aimed at explaining the rationale of therapy and WES2 to designate interventions where the therapist frames the clinical setting by encouraging the patient to express their thoughts freely during the session. Clarification (CL) could be categorised as CL1, referring to interventions by the therapist aimed at summarising what the patient has said, while CL2 pertains to interventions that rephrase the patient's statements to provide a more integrated narrative without offering an interpretation. Associations could be classified as ASS1 for therapists' general statements of fact or opinion, ASS2 for therapeutic statements concerning events previously reported in therapy (e.g., in a previous session) and ASS3 for therapist self‐disclosures. These distinctions arise from the crucial role of therapist self‐disclosure interventions, particularly in enhancing positive qualities related to outcomes such as fostering the therapeutic relationship/alliance (Henretty and Levitt 2010). Particularly, our suggestion is to refer to self‐disclosure as defined by McCarthy and Betz (1978), in terms of *‘self‐disclosing’* disclosures, that is, when the therapist shares a personal experience that does not directly relate to the client, rather than instances where the therapist expresses their immediate feelings or reactions to the client (i.e., *‘self‐involving’* disclosure).

Exploring the use of PIRS, and consequently its strengths and limitations, is relevant in the context of psychotherapy research, which is increasingly moving towards opening the ‘black box’ (Kleinbub, Talia, and Palmieri 2020) of psychotherapy sessions, going beyond outcome results. Further detailing a measure already widespread and transversal to approaches such as PIRS contributes to enhancing the depth of investigation of the therapeutic micro‐process by researchers, also to enter the arena of the emerging field of interpersonal physiology, whose underlying functioning of the micro‐process represents one of the greatest challenges in understanding research in psychotherapy.

### 4.6 Limitations of the Reviewed Literature

The articles included in this systematic review are relatively few and encompass diverse theoretical models, such as narrative group counselling (e.g., Esposito, Marano, and Freda 2018; Esposito, Karterud, and Freda 2021) and STDP (Bhatia et al. 2016, 2019; Gerostathos et al. 2014; Petraglia et al. 2015, 2018; Sahli, de Roten, and Despland 2015). Additionally, many of the included studies are somewhat outdated, with 14 out of 22 conducted between 1998 and 2015. In addition, it is worth noting that the samples included are limited by small sizes and restricted to specific populations, such as students or specific clinical populations (e.g., Banon, Evan‐Grenier, and Bond 2001; Bond, Banon, and Grenier 1998; Sahli, de Roten, and Despland 2015), which jeopardises the generalisability of the results. Finally, some of these works also exhibit methodological concerns, such as correlational designs and macro‐analytic level analysis (e.g., Bhatia et al. 2019; Gerostathos et al. 2014; Petraglia et al. 2015) or methodology susceptible to statistical power issues (e.g., Petraglia et al. 2018). Further future developments regarding a thorough examination of the use of the PIRS and other coding systems based on verbal transcripts could utilise a method of analysis that has already been used in Colli, Gagliardini, and Gullo (2022) and is increasingly adopted in clinical psychology (e.g., Gullo et al. 2023), that is, multiple mediation analysis. This would allow for considering how concurrent clinical phenomena (e.g., the quality of the therapeutic alliance and the patient's defence mechanisms) influence or mediate the therapist's interventions. Meta‐analyses should also be an appropriate tool to further investigate the role of the PIRS in capturing psychotherapeutic microprocesses.

## 5 Conclusions

The examined literature indicates that therapists should maintain an awareness of their interventions and their peculiar associations with patients' defensive functioning and the quality of the therapeutic alliance. Overall, the results of this systematic review have shown that, due to their contribution to strengthening the therapeutic alliance, Supportive Interventions are especially valuable in the early stages of therapy, as they lay the groundwork for more interpretive techniques, which are optimally introduced once a solid therapeutic alliance has been established. This consideration is particularly central in the case of ‘high‐risk, high‐gain’ interventions with an alliance‐building impact, such as Transference interpretations (Banon, Evan‐Grenier, and Bond 2001; Bond, Banon, and Grenier 1998). Furthermore, as revealed by the studies that utilised indices derived from the PIRS (i.e., TAD, IA, ACU, CFL and AR), it is critical for therapists to remain attuned to patients and accurately adapt their interventions to the patients' level of defensive functioning and also ensure that their Interpretive interventions are aligned with the patients' central relational themes and experiential meanings, as this strengthens the therapeutic alliance, leading to better therapeutic outcomes. Additionally, the authors emphasise that therapists should remain sensitive to their countertransference, particularly when they experience reactive responses and avoid offering interpretations driven by these reactions. Such awareness becomes especially important during moments of therapeutic impasse, as Interpretive interventions may serve as a defence mechanism on the therapist's part rather than facilitate patient change. They may also represent the therapist's attempt to cope with the therapeutic situation. As emerged from the literature review (Romano et al. 2009), therapists should also be cognizant of the clinical implications of colluding with patients' maladaptive relational patterns and how this can influence their interventions, potentially leading to more directive techniques in the therapeutic discourse.

The findings of this systematic review align with the observations made by Gabbard (2014), indicating that an effective therapeutic approach is neither purely supportive nor purely expressive. Expressive and Supportive Interventions are indeed conceived to be used alternately within therapy, depending on the patient's level of organization, personality traits, predominant defence mechanisms and ego strength (Gabbard 2014; Luborsky 1984). Overall, the PIRS is confirmed to be a highly valid and effective tool for understanding the micro‐process, and it may benefit from a more refined revision concerning specific categories, which could further enhance its utility in understanding the therapeutic process.

## Conflicts of Interest

The authors declare no conflicts of interest.

## Data Availability

The data supporting this systematic review are derived from publicly available studies identified through systematic searches as described in the PRISMA flow diagram. No new data were generated for this study.
